# The role of the MAPK pathway alterations in GM-CSF modulated human neutrophil apoptosis with aging

**DOI:** 10.1186/1742-4933-2-6

**Published:** 2005-03-02

**Authors:** Anis Larbi, Nadine Douziech, Carl Fortin, Annie Linteau, Gilles Dupuis, Tamas Fulop

**Affiliations:** 1 Laboratory of Immunology, Research Center on Aging, University of Sherbrooke, Qc, Canada; 2 Immunology Graduate Program, Clinical Research Center, Faculty of Medicine, University of Sherbrooke, Qc, Canada; 3 Signal Transduction Laboratory, Department of Biochemistry, University of Sherbrooke, Qc, Canada; 4 Department of Medicine, Geriatrics Division, Faculty of medicine, University of Sherbrooke, Qc, Canada

**Keywords:** Neutrophils, apoptosis, aging, GM-CSF, MAPK pathway

## Abstract

**Background:**

Neutrophils represent the first line of defence against aggressions. The programmed death of neutrophils is delayed by pro-inflammatory stimuli to ensure a proper resolution of the inflammation in time and place. The pro-inflammatory stimuli include granulocyte-macrophage colony-stimulating factor (GM-CSF). Recently, we have demonstrated that although neutrophils have an identical spontaneous apoptosis in elderly subjects compared to that in young subjects, the GM-CSF-induced delayed apoptosis is markedly diminished. The present study investigates whether an alteration of the GM-CSF stimulation of MAPKs play a role in the diminished rescue from apoptosis of PMN of elderly subjects.

**Methods:**

Neutrophils were separated from healthy young and elderly donors satisfying the SENIEUR protocol. Neutrophils were stimulated with GM-CSF and inhibitors of the MAPKinase pathway. Apoptosis commitment, phosphorylation of signaling molecules, caspase-3 activities as well as expression of pro- and anti-apoptotic molecules were performed in this study. Data were analyzed using Student's two-tailed *t*-test for independent means. Significance was set for p ≤ 0.05 unless stated otherwise.

**Results:**

In this paper we present evidence that an alteration in the p42/p44 MAPK activation occurs in PMN of elderly subjects under GM-CSF stimulation and this plays a role in the decreased delay of apoptosis of PMN in elderly. We also show that p38 MAPK does not play a role in GM-CSF delayed apoptosis in PMN of any age-groups, while it participates to the spontaneous apoptosis. Our results also show that the alteration of the p42/p44 MAPK activation contributes to the inability of GM-CSF to decrease the caspase-3 activation in PMN of elderly subjects. Moreover, GM-CSF converts the pro-apoptotic phenotype to an anti-apoptotic phenotype by modulating the bcl-2 family members Bax and Bcl-xL in PMN of young subjects, while this does not occur in PMN of elderly. However, this modulation seems MAPK independent.

**Conclusion:**

Our results show that the alteration of p42/p44 MAPK activation contributes to the GM-CSF induced decreased PMN rescue from apoptosis in elderly subjects. The modulation of MAPK activation in PMN of elderly subjects might help to restore the functionality of PMN with aging.

## Introduction

Neutrophils represent the first line of defence against aggressions [1]. They are the first cells to arrive at the site of the aggression. Neutrophils can directly eliminate the invading organisms, but most of the time they set the stage, with other cells of the innate immune system including macrophages and dendritic cells, to the development of the adaptive immune response [2]. The interaction between the innate and adaptive immune response confer to the organism an efficacious defence against infections, cancers and other aggressions.

Once the neutrophils finished their cleaning and modulating role, they should disappear in an ordered manner without releasing toxic products from their granules that eventually harmed the surrounding tissues. If they do not disappear they would induce a chronic inflammatory process. Their elimination for the sake of the organism is occurring through apoptosis [3-5]. Thus, neutrophils are programmed to die spontaneously in the absence of pro-inflammatory stimuli [6,7]. This death assures a proper resolution of the inflammation in time and place.

However, in the presence of pro-inflammatory stimuli including lipopolysaccharide (LPS), granulocyte-colony-stimulating factor (G-CSF), granulocyte-macrophage colony-stimulating factor (GM-CSF) neutrophils are able to postpone their spontaneous propensity for dying and thus, remain active during more than 72 hours [8]. This delayed apoptosis confers to neutrophils a highly efficacious manner to maintain their activity be able to eliminate properly the aggressors.

It is well accepted that aging is linked to an increase in the susceptibility to various infections [9]. This is mostly related to the dysregulation of the immune response [10-12]. The most studied part is the T-cell induced cellular immune response, which is considered to be the most affected by the aging process. However, nowadays it is also accepted that neutrophils functions are also altered with aging, even in healthy elderly satisfying the SENIEUR protocol requirements [13-15]. The most affected functions are the chemotaxis, the free radical productions and killing. Recently, we have demonstrated that although neutrophils have an identical spontaneous apoptosis in elderly subjects compared to that in young subjects, the GM-CSF induced delayed apoptosis is markedly diminished [6]. This observation was confirmed by other groups [17,18]. This decreased GM-CSF induced delay in PMN apoptosis could have far reaching consequences for PMN functions and the body defence against infections.

The mechanism of the GM-CSF induced delay of PMN apoptosis is under intense investigation. It is well known that GM-CSF induces three distinct signalling pathways in neutrophils: the Jak/STAT, the MAPK and the PI3K pathways [19-21]. Recently, it became evident that the MAPK and PI3K pathways are participating in the GM-CSF induced delayed apoptosis of PMN in young subjects [22]. Recently, we provided evidence that the Jak/STAT pathway is also contributing to the apoptosis delaying effect of GM-CSF (manuscript submitted). Furthermore, these signalling pathways modulate the expression of Bcl-2 family members as well as that of caspases which play a determinant role in the fate of cells towards survival or apoptotic death [23-25].

MAPKinases have been shown to have important roles in intracellular mechanisms responsible for neutrophils activation induced by various stimuli, as well as the modulation of their apoptosis [23,26-30]. There exist three different MAPKs: the extracellular regulated kinase (ERK1/2 or p42/44), the p38 and the c-Jun terminal kinase (JNK). Activation of the p42/44 MAPK occurs through phosphorylation of threonine and tyrosine residues by an upstream MAPKK (MEK1 and 2) [31]. Both kinases are known to be weakly auto-phosphorylated on tyrosine. The activation of p38 is occurring in the same manner with the MEK3/6 [32]. The p42/p44 MAPK is definitively involved in the PMN apoptosis rescuing activity of various agents including LPS, GM-CSF [26-30] while the role of p38 MAPK remains controversial and seems to depend on the stimuli used [33-36].

The present study investigates whether an alteration of the GM-CSF MAPK stimulation play a role in the diminished rescue from apoptosis of PMN of elderly subjects. In the present paper we present evidence that an alteration in the ERK1/2 activation occurs in PMN of elderly subjects under GM-CSF stimulation and this plays a role in the decreased delay of apoptosis of PMN in elderly. We also show that p38 MAPK does not play a role in GM-CSF delayed apoptosis in PMN of any age-groups. Our data also show that the alteration of the p42/p44 MAPK activation results in the inability of GM-CSF to convert the pro-apoptotic phenotype of PMN of elderly subjects to an anti-apoptotic phenotype by modulating the Bcl-2 family members Bax and Bcl-xL as well as the caspase-3.

## Materials and methods

### Reagents and antibodies

Human recombinant GM-CSF was purchased from Calbiochem-Novabiochem (La Jolla, CA). Ethylene glycol-bis (β-aminoethyl ether)-*N, N, N', N'*-tetraacetic acid (EGTA), aprotinin, sodium orthovanadate (Na_3_VO_4_), phenylmethylsulfonyl fluoride (PMSF) and ethylenediaminetetraacetic acid (EDTA) were obtained from Sigma-Aldrich (St Louis, MO). Leupeptin, chymostatin and pepstatin were from Boehringer Mannheim (Mannheim, Germany). Iscove's medium was purchased from Life Technologies (Grand Island, NY). The anti-caspase-3 antibody recognizing the p17 fragment of cleaved caspase-3 was a generous gift of Dr. D. Nicholson (Merck Frosst Co., Montreal, QC). Anti-phosphotyrosine mAb (4G10), anti-Bax and anti-Bcl-xL were purchased from Upstate Biotechnology Inc. (Lake Placid, NY). Polyclonal anti phospho-p42/p44 MAPK (Thr202/Tyr204), anti-p42/p44 MAPK, anti phospho-p38 MAPK (Thr180/Tyr182) and anti-p38 MAPK antibodies were from Santa Cruz (Santa Cruz, CA). The MEK inhibitor PD98059 and p38 inhibitor SB203580 were from Calbiochem. Cell permeable inhibitors of caspase-3 (Z-DEVD-FMK) and caspase-8 (Z-IETD-FMK) were purchased from Bio-Rad Laboratories (Mississauga, ON). Fluorometric caspase-3 substrate (Ac-DEVD-AMC), caspase-3 inhibitor (DEVD-CHO), caspase-8 substrate (Ac-IETD-AMC) and caspase-8 inhibitor (IETD-CHO) were from Biosource International. Propidium iodide was from R&D Systems (Minneapolis, MN). Other reagents were obtained from Sigma-Aldrich unless stated otherwise.

### PMN isolation

Venous blood was collected from 20 young (20–25 years) and 20 elderly (65–85 years) individuals satisfying the SENIEUR protocol criteria for immunogerontological studies, as described [37]. All subjects gave their informed consent and the protocol was approved by the Hospital Ethical Committee. Neutrophils were separated by sequential sedimentation on 2% Dextran T-500 (Amersham Biosciences) in 0.9% sodium chloride, centrifugation on a Ficoll-Hypaque cushion (specific gravity 1.077, Amersham Health, Baie d'Urfé, QC) and hypotonic lysis of erythrocytes, as described [16]. Light microscopy showed that more than 97% of the cell population was composed of neutrophils. Cell viability was greater than 95% as assessed by Trypan blue exclusion.

### PMN cultures

All experiments were performed using media, serum and reagents that were free of endotoxins to avoid non-specific activation of PMN. Purified PMN were suspended (5 × 10^6 ^cells/ml) in Iscove's modified Dulbecco's medium supplemented with 10% autologous serum, 50 U/ml penicillin and 25 μg/ml streptomycin and incubated in the presence or absence (control) of GM-CSF, at a concentration of 20 ng/ml which had been previously determined to induce maximal PMN response. Other agents used included: H_2_O_2 _(250 μM) and FMLP (10^-8 ^M). These concentrations were previously determined to induce maximal activation (16). The cells were incubated for various periods of time (6 and 18 hours) in polypropylene tubes (Becton Dickinson Labware, Lincoln Park, NJ) at 37°C in a humidified 5% CO_2_-95% air incubator. For some experiments the MEK1/2 inhibitor, PD98059, and the p38 inhibitor, SB203580, were used at 30 μM and 10 μM, respectively for 1 hour prior to GM-CSF stimulation, as this concentration (from 7,5 to 100 μM) and time point (from 30 to 240 min) had been shown to be the most effective to block these MAPK activities. Stock solution of GM-CSF, H_2_O_2_, FMLP, and PD98059 as well as SB203580 were prepared in DMSO (final concentration < 0.01% v/v). Preliminary experiments showed that these concentrations of DMSO did not increase cell necrosis or the rate of PMN apoptosis.

### Assessment of PMN apoptosis

Apoptosis of PMN was measured by flow cytometry using FITC-conjugated Annexin V to label externalized phosphatidylserine, whereas propidium iodide staining was used to differentiate apoptosis from necrosis [38]. The cells were washed in cold PBS and then gently resuspended in 0.5 ml of binding buffer (10 mM HEPES, 140 mM NaCl, 2.5 mM CaCl_2_, pH 7.4) in 12 × 75 mm polystyrene tubes. FITC-Annexin V at a saturating concentration was added to the cell suspension and incubations were performed in the dark at room temperature for 15 min. Non-specific staining was determined using a Ca^2+^-free buffer (10 mM HEPES, 140 mM NaCl, 10 mM EDTA, pH 7.4). Fluorescence was filtered through a 530/30 nm band pass to record FITC-Annexin V emission and a 582/42 nm band pass to detect PI emission. Fluorescence intensity was measured on a FACScan flow cytometer (Becton Dickinson, Mountain View, CA) equipped with a 15 mm air cooled 488 nm argon-ion laser. Gating on physical parameters was used to exclude cell debris and clumps. A minimum of 10,000 events was analyzed in each experiment.

### Western blot analysis of p42/p44 MAPK, p38 MAPK, Bax, Bcl-xL and caspase-3

PMN (10^7 ^cells) were cultured in the absence or presence of GM-CSF (20 ng/ml) and lysed in a buffer containing 20 mM Tris-HCl, pH 7.4, 137 mM NaCl, 10% glycerol, 1% Nonidet P-40, 2 mM sodium vanadate and 100 mM sodium fluoride for a 30 min on ice. Cell lysates were centrifuged at 16,000 × *g *for 15 min and protein concentration of the supernatants was determined by using the Bradford protein assay reagent (Bio-Rad). 20 μg of cell lysates were resolved on a 8% SDS-PAGE, transferred to Hybond nitrocellulose membranes (Amersham Biosciences) and antigens revealed by probing the membrane with an anti-phosphotyrosine antibody (4G10) or the relevant antibodies p42/p44MAPK and p38MAPK (phosphorylated or not), Bax, Bcl-xL, caspase-3. Membranes were then washed six times with TBS and incubated with horseradish peroxidase-conjugated secondary antibody for 1 h at room temperature. The protein bands were revealed by densitometry analysis was performed using the Chemigenius^2 ^Bio Imaging System (Syngene, Frederick, MD) [37].

### Determination of caspase 3-activity

PMN (5 × 10^6 ^cells) were lysed in 100 μl of a 10 mM potassium phosphate, 1 mM EDTA buffer (pH:7.4) containing 0.5% Triton X-100 supplemented with 2 mM PMSF, 10 μg/ml leupeptin, 10 μg/ml pepstatin, and 10 mM dithiotreitol for 15 min on ice and spun at 14,000 rpm for 20 min. The lysate (100 μg) was diluted to 1 ml with ICE buffer (50 mM HEPES, 10% sucrose, 0.1% CHAPS, pH 7.5) containing 50 μM of the caspase-3 substrate Ac-DEVD-AMC (aspartate-glutamate-valine-aspartate-AMC) [23] and 10 mM freshly prepared dithiotreitol. Five hundred μl of reaction mixture were diluted with 1.5 ml of ICE buffer and fluorescence (excitation wavelength 400 nm, emission wavelength 505 nm) was read at 1 h time point. The release of fluorochrome was linear with time and with the protein concentration used. Standards containing 0–1500 nM AMC were used to determine the amount of spontaneous release of fluorochrome. Specificity for caspase-3 activity was demonstrated by using the caspase-3 inhibitor DEVD-CHO (24). A very low level of non-specific activity was present, and the effects of the inhibitor were concentration-dependent. The inhibitor of caspase-8 IETD-CHO [25] activity assessed by the fluorochrome substrate Ac-IETD-AMC was also included to insure the specificity of caspase-3 activation.

### Statistical analysis

Results are representative of experiments performed with 20 individual donors of each age group. Results are presented as pooled data from the entire series of experiments (mean ± SD). Data were analyzed using Student's two-tailed *t*-test for independent means. Significance was set for p ≤ 0.05 unless stated otherwise.

## Results

### 1. Spontaneous and GM-CSF-induced apoptosis of PMN: effect of aging

#### 1.1 Spontaneous apoptosis

**Figure 1:**
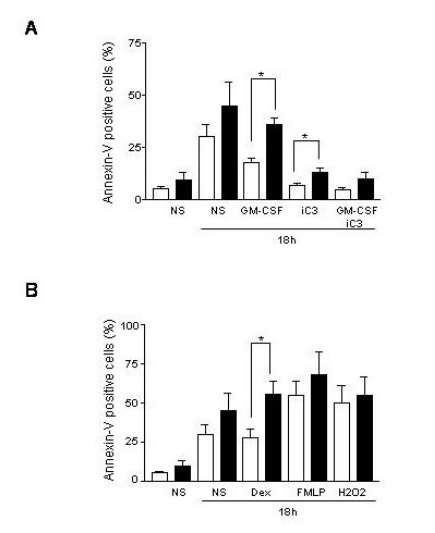
**Measurement of human neutrophils apoptosis with aging by flow cytometry. A**) GM-CSF-treated (20 ng/ml) or untreated neutrophils were cultured during various time periods (from t = 0 to t = 18 h) and stained with Annexin-V conjugated to FITC. FACS analyses were performed for fluorescence intensity and data were reported in a time-dependent manner for the different experimental conditions and compared to control neutrophils from young (white columns) and elderly donors (black columns). Identical experiments were done with Z-DEVD-FMK, inhibitor of caspase-3 activity (iC3) in the presence or absence of GM-CSF. Data represent percentage of apoptotic cells from the neutrophil pool. Data were statistically analyzed for 20 different donors of each group indicated by *p < 0.01. **B**) The same experiments were done using H_2_O_2 _(250 μM), Dexamethasone (10^-8 ^M) and FMLP (10^-8 ^M). Data are from 10 independent experiments with *p < 0.01.

In control neutrophils obtained from young subjects, the mean percentage of apoptotic cells increased steadily from 5 ± 2.7% at 0 hour to 30 ± 6.5% at 18 hours (Fig 1A and 1B, white columns, NS). Similar observations were found for PMN of elderly subjects where we found that 8 ± 3.0%, 14 ± 3.2% and 44 ± 7.0% were apoptotic, measured by the expression of Annexin-V at 0 h and 18 h, respectively (Figure 1A, filled columns, NS). Even if a tendency towards a higher susceptibility to apoptosis is observed with aging, this is not significant. It is of note that, the differences in PMN apoptosis between the basal state and after 18 h of culture are statistically significant in case of PMN of young (p < 0.01), as well as of elderly (p < 0.01) subjects.

#### 1.2 Effects of various stimulants on apoptosis of neutrophils cultured in vitro

We evaluated, by the expression of Annexin-V using flow cytometry measurements, the effects of GM-CSF, H_2_O_2_, Dexamethasone (Dex), FMLP and Z-DEVD-FMK, an inhibitor of caspase-3 (iC3) on the modulation of the PMN apoptosis for various periods of time. It is well known that GM-CSF is able to rescue PMN from their spontaneous apoptosis [6]. As shown in Figure 1A the percentage of apoptotic PMN of young subjects treated by GM-CSF decreased significantly (p < 0.01) after 18 hours of culture compared to control cultures, starting already at 6 hours (data not shown). In contrast, in PMN of elderly subjects after GM-CSF treatment a decrease in apoptosis starting at 6 hours of culture could be also observed, however this did not become statistically significant at any culture time (at 18 hours) compared to control cultures (Figure 1A, filled columns). It is of note that no age-related differences could be demonstrated for the other treatments (Figure 1B), except for dexamethasone. In contrast to GM-CSF, dexamethasone did not decrease significantly the PMN apoptosis in young subjects, but slightly increased that of elderly subjects, which resulted in a significant difference with age (Figure 1B). Finally, we studied the effect of an inhibitor of caspase-3 on PMN spontaneous apoptosis. We found that the iC3 significantly decreased the spontaneous apoptosis of PMN in both age-groups (Figure 1A). The caspase-3 inhibitor significantly increased the anti-apoptotic effect of GM-CSF as compared to GM-CSF alone in PMN of young subjects (5 % vs 14 %, respectively; p < 0.05). These results indicate an efficient synergism at 18 h between the effect of GM-CSF and of the caspase-3 inhibitor when compared to spontaneous apoptosis in PMN of young subjects (5 % vs 30 %, respectively; p < 0.05). The synergism seen in the case of young donors is missing in the case of elderly donors since GM-CSF is inefficient. Furthermore, these results indicate that caspase-3 is implicated in the spontaneous and GM-CSF modulated apoptosis of PMN of young and elderly subjects. Altogether, these results suggested that GM-CSF sustained survival is defective with aging. Thus, the question rose, whether an alteration in the signal transduction of GM-CSF receptor could contribute to the altered rescue of PMN of elderly subjects from apoptosis.

### 2. Study of the involvement of the MAPK signalling pathways in the GM-CSF induced rescue from apoptosis of PMN of young and elderly subjects

**Figure 2:**
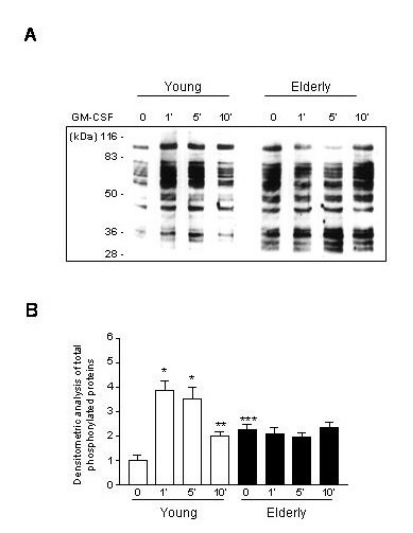
**Protein tyrosine phosphorylation of whole PMN cell lysates after short stimulation with GM-CSF **Freshly prepared neutrophils were stimulated with GM-CSF for 1 to 10 min and immediately lysed. Lysates were sized on SDS-PAGE followed by western-blotting using anti-phosphotyrosine mAbs to reveal protein phosphorylation. The gel shown here is representative of 10 independent experiments. Image analyzer was used to measure the amount of phosphorylation and represented above the gel for each experimental condition in the case of young (white columns) and elderly donors (black columns). Similar phosphorylation patterns were obtained from others experiments. A significant increase in total phosphorylation in indicated by *p < 0.01, **p < 0.05. A significant difference in basal phosphorylation status of PMN from young and elderly donors was found and indicated by ***p < 0.05.

PMN of young and elderly subjects were stimulated with GM-CSF during various periods of time (1, 5 and 10 minutes, see figure 2 lanes 2, 3, 4), cytosolic proteins were sized by SDS-PAGE and their pattern of tyrosine phosphorylation was assessed by Western blotting with an anti-phosphotyrosine mAb (4G10). At the basal level, the protein-tyrosine phosphorylation was significantly already enhanced in PMN of elderly subjects compared to that of young subjects (Figure 2A lane 1, p < 0.01). There is a kinetic increase of the protein-tyrosine phosphorylation of almost each bands (e.g. p42/44, p110) in PMN of young subjects after GM-CSF stimulation at 1 min and 5 min (lane 2 and lane 3 respectively, left panel, p < 0.01 compared to basal state) starting to decrease at 10 min (lane 4 left panel). In contrast, no significant kinetics in the protein-tyrosine phosphorylation could be observed in PMN from elderly subjects stimulated with GM-CSF, probably because of the intense protein-tyrosine phosphorylation level at the basal state (Figure 2A lane 1 right panel). Figure 2B is representing the densitometric scanning of the gels as described in the Materials and Methods section.

**Figure 3:**
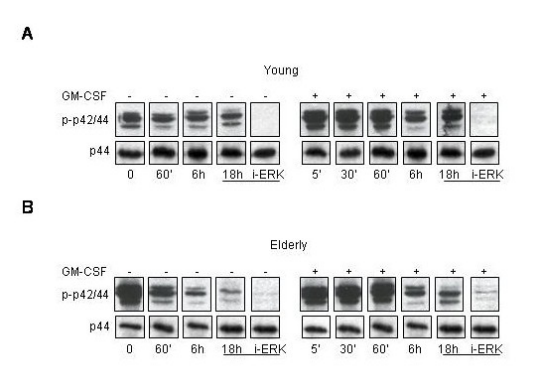
**Activation of p42/p44 MAPK following short and long-time incubation with GM-CSF, effect of aging. **Neutrophils of young (**A**) and elderly subjects (**B**) were stimulated either for 5, 30, 60 min, 6 and 18 hours with GM-CSF alone or pre-treated with PD98059 (i-ERK) for 1 hours prior to GM-CSF stimulation for 18 hours. Neutrophils were then lysed and analyzed by western-blotting experiments for p42/p44 MAPK phosphorylation revealing by phospho-anti-p42/p44 MAPK polyclonal Abs. Control loading are shown under each blot for p44. Experiments were performed for 15 different donors of each group.

We then focused on p42/p44 MAPK proteins which were shown to be activated in PMN following GM-CSF receptor stimulation, as well as by other stimulants [26-30]. It was already demonstrated that p42/p44 MAPK activation contributed to the decrease of spontaneous apoptosis by GM-CSF [22]. Therefore, we studied the GM-CSF induced ERK1/2 activation in PMN in both age-groups. It is of note that the ERK1/2 tyrosine phosphorylation already detectable at basal level did not change during the non-stimulated culture times (1 h, 6 h, 18 h) when compared to the protein level expression. However, the pre-incubation of the PMN of young subjects with an inhibitor of MEK1/2, PD98059 (i-ERK), lead to the complete abrogation of p42/p44MAPK phosphorylation after 18 hours (Figure 3, young subjects, left panel). In PMN of young subjects the protein-tyrosine phosphorylation of p42/p44MAPK increased by two times after 5 minutes of GM-CSF stimulation and remained thereafter for 18 hours with a slight decrease at 6 hours (Figure 2, young subjects, right panel). This increase was mainly due to the p44 components of the ERK1/2. Once more the PD98059 abrogated the GM-CSF induced ERK1/2 activation measured by tyrosine phosphorylation. In contrast, in PMN of elderly we observed a very strong basal tyrosine phosphorylation (non-stimulated) compared to that of young subjects (p < 0.01), which decreased thereafter during the non-stimulated incubation (Figure 3, elderly subjects, left panel). We could not demonstrate any changes in tyrosine phosphorylation of p42/p44MAPK during the 6 first hours activation by GM-CSF in PMN of elderly donors (Figure 3, elderly subjects, right panel), which even decreased thereafter during the 18 hours of stimulation. The i-ERK could only partially inhibit the GM-CSF induced ERK1/2 tyrosine phosphorylation. Altogether these data indicate that GM-CSF activates the p42/p44 MAPK by phosphorylation on tyrosine in a sustained manner during the 18 hours of culture in PMN of young, while this activation is completely absent in PMN of elderly subjects.

**Figure 4:**
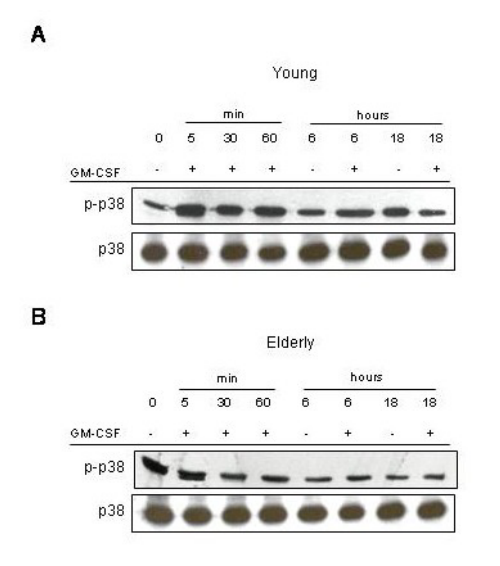
**Time-dependant activation of p38 MAPK following incubation with GM-CSF, effect of aging. **Neutrophils were stimulated with GM-CSF from 0 to 18 hours and immediately lysed. Western-blotting experiments of neutrophils of young (**A**) and elderly subjects (**B**) lysates were conducted by revealing with anti-phospho-p38 MAPK polyclonal Abs. Control loading for p38 are shown under each blot. Blots are representative of 15 independent experiments.

Next, we assessed the protein tyrosine phosphorylation of p38 MAPK (Figure 4). The role of p38 in PMN apoptosis is still controversial [33-36]. There is no evidence that GM-CSF modulate its activation. However, p38 MAPK activation has been implicated in the spontaneous apoptosis of PMN [33,34]. We present here data that p38 MAPK is tyrosine phosphorylated already at the basal level, in a significantly (p < 0.01) higher level in PMN of elderly subjects compared to young subjects (Figure 4A and 4B). We also show an increase in p38 MAPK phosphorylation after 5 min of stimulation by GM-CSF in PMN of young subjects sustained for 6 hours (Figure 3A, p < 0.01) and returning to the basal level after 18 hours when comparing with control protein loading. It is of note than when the PMN were left untreated for 18 hours the phosphorylation of p38 on tyrosine was significantly increased either compared to the basal non-stimulated status (p < 0.05) or to the 18 hours GM-CSF stimulation (p < 0.05). There was no tyrosine phosphorylation modulation of p38 in PMN of elderly subjects in response to GM-CSF stimulation (Figure 3B), which was already very high at basal level compared to PMN of young subjects (p < 0.01). These data also suggest that p38 MAPK participate to the spontaneous apoptosis of PMN in both age-groups, but did not participate to the GM-CSF delay of apoptosis. These results further indicate that we assist to an alteration of the signal transduction in PMN of elderly subjects in regard to the MAPK pathways.

Then, we tried to link the alteration described previously in the MAPK pathways in PMN of elderly subjects to the inability of GM-CSF to rescue them from apoptosis using the pharmacological inhibitors of p42/p44 and p38 MAPKs. We assessed the spontaneous and GM-CSF delayed apoptosis of PMN after 18 hours treatment with PD98059 and SB203580 (Table 1). We found by Annexin-V/PI staining that apoptosis commitment was not significantly affected by any of these inhibitors in PMN of young and elderly subjects (Table 1). When the PD98059 was applied as pre-treatment to GM-CSF stimulation, it completely abolished the GM-CSF anti-apoptotic effect in PMN of young subjects, while it had no effect on GM-CSF of elderly subjects (Table 1). When the SB203580 was applied as a pre-treatment prior to GM-CSF stimulation it could not abrogated the GM-CSF apoptosis rescuing effect, indicating that p38 is not concerned by the GM-CSF induced rescue. In PMN of elderly, the SB203580 pre-incubation had a similar effect than in PMN of young individuals. Altogether, these data revealed two main observations, first, the role of the p42/p44 MAPK pathway in PMN survival in contrast to that of p38 MAPK under GM-CSF stimulations and second, the altered stimulating effect of GM-CSF via the decreased p42/p44 MAPK phosphorylation due to an increased basal over-activation contributes to the decreased survival of PMN of elderly subjects.

**Table 1:** Neutrophil apoptosis modulation by MAPKinase inhibitors

Treatment	Culture time (hours)	Annexin-V positive cells (%)	
		
		Young	Elderly
			
	0	11 ± 2	15 ± 3
			
None	6	11 ± 3	20 ± 3
	18	34 ± 6	48 ± 10
			
GM-CSF	6	11 ± 4	10 ± 5
	18	18 ± 3	41 ± 9*
			
PD98059	18	33 ± 7	45 ± 10
			
GM-CSF + PD98059	18	40 ± 10	50 ± 15
			
SB203580	18	43 ± 2	53 ± 5*
			
GM-CSF + SB203580	18	24 ± 3	33 ± 5*

The cells were cultured for the indicated time in the presence or absence of GM-CSF (20 ng/ml) and the presence or absence of PD98059 (30 μM) or SB203580 (10 μM). The percentage of apoptotic was determined by labeling with FITC-conjugated Annexin-V and cell integrity, by Propidium iodide (PI) staining. The cells were analyzed by cytofluorimetry. Data are shown as the mean ± SD of five 5 independent experiments. Significant differences between young and elderly subjects are indicated by an asterisk (*, p < 0.05 and **, p <0.01).

### 3. Role of the Bcl-2 family members Bax and Bcl-xL in GM-CSF induced rescue of PMN from apoptosis

**Figure 5:**
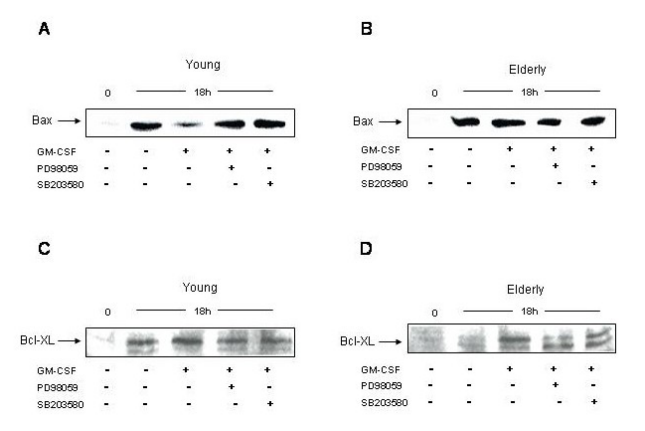
**Expression of bcl-2 family members Bax and Bcl-xL in PMN with aging upon treatment with GM-CSF and PD98059 and SB203580 for 18 h. **Neutrophils were cultured in presence of GM-CSF alone or in combination with either the p42/p44 MAPK inhibitor, PD98059, or the p38 MAPK inhibitor, SB203580, for 18 h. Then, neutrophils were lysed and western-blotting experiments were executed to investigate the amount of Bax in young (**A**) and elderly donors (**B**). The same experiments were conducted for studying Bcl-xl expression in PMN of young (**C**) and elderly subjects (**D**). GM-CSF significantly decreased the expression of Bax in PMN of young subjects (A, p < 0.01), without any effect in PMN of elderly subjects (B). GM-CSF significantly increased the expression of Bcl-xL (p < 0.05) in both age groups (C and D), Blots are representative of 15 independent experiments.

**Figure 6:**
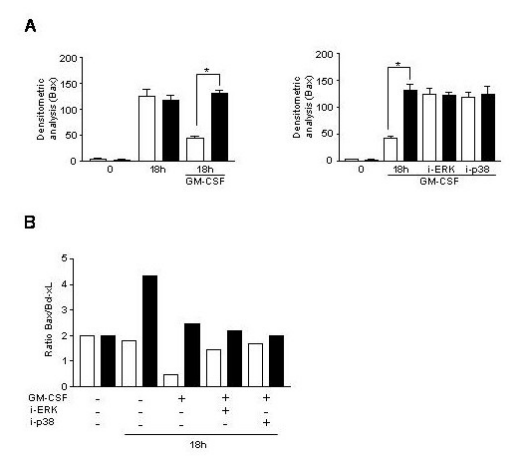
**Densitometric analyses of the expression of bcl-2 family member Bax in PMN with aging upon treatment with GM-CSF and PD98059 and SB203580 for 18 h as well as the ratio of the expression of Bax/Bcl-xL. **Densitometric analysis were performed as described in the Materials and Methods section for the expression of Bax in neutrophils of young (white columns) and elderly subjects (black columns) under GM-CSF stimulation (**A**, *p < 0.01) and after the application of inhibitors, PD98059 (i-ERK) and SB203580 (i-p38) (**B**, p < 0.01). The ratio of Bax to Bcl-xL in PMN of young (white columns) and elderly subjects (black columns) under GM-CSF stimulation and modulation by inhibitors is represented (**C**).

The Bcl-2 family members are actively participating in the apoptotic process by being either pro-apoptotic such as Bax or anti-apoptotic such as Bcl-xL [6]. Their role in the PMN spontaneous and GM-CSF induced apoptosis was recently studied [9]. It was shown that the ratio of the pro- and anti-apoptotic molecules was essential. However, the presence of Bcl-xL in PMN is still debated [40]. Here, first we studied how the GM-CSF affected Bax and Bcl-xL expression after 18 hours of culture in PMN of young and elderly subjects. We found that in PMN of young subjects concomitantly to its anti-apoptotic effect the GM-CSF decrease significantly the expression of Bax (p < 0.01) and increase the expression of Bcl-xL (Figure 5A and 5C, Figure 6A). In PMN of elderly subjects the GM-CSF was unable to modulate the expression of Bax, however slightly increased that of Bcl-xL (Figure 5B and 5D, Figure 6A). Nevertheless, when the ratio is calculated there is a significant shift towards survival (Bcl-xL) in PMN of young subjects (Figure 6B), while this is the contrary in PMN of elderly (increase of Bax) (Figure 6B). When the inhibitors were used they did not modulate the expression neither of Bax nor of Bcl-xL in any age-groups. These data altogether indicate that the GM-CSF stimulation create an anti-apoptotic milieu in the ratio of the Bcl-2 pro-and anti-apoptotic members, while this is the contrary in PMN of elderly subjects. Moreover, the MAPK pathways do not seem to intervene in the modulation of the expression of the Bcl-2 family members in PMN.

### 4. Role of caspase-3 in the apoptosis of PMN and its modulation by GM-CSF through the MAPK pathway

**Figure 7:**
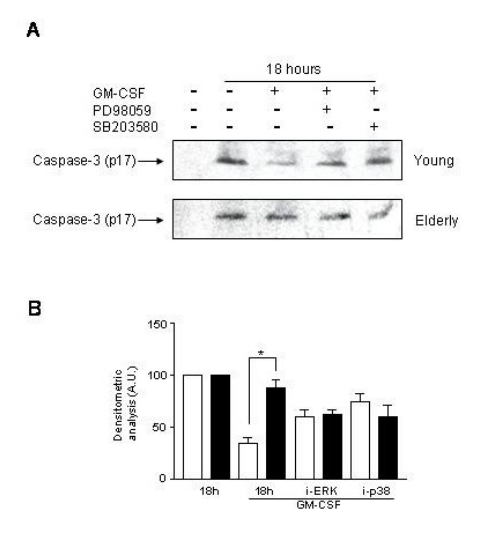
**Expression of activated caspase-3 in PMN with aging upon treatment with GM-CSF and PD98059 and SB203580 for 18 h A**) Neutrophils of young (upper panel) and elderly subjects (lower panel) were cultured in presence of GM-CSF alone or in combination with either the inhibitor PD98059 (i-ERK) or SB203580 (i-p38), for 18 h. Then, neutrophils were lysed and western-blotting experiments were performed to investigate the amount of active caspase-3. With aging no changes were found by any agents used (lower panel) while neutrophils from young donors (upper panel) showed significant modulation by GM-CSF and PD98059. **B**, Densitometric analyses were performed as described in the Materials and Methods section for the expression of activated caspase-3 in neutrophils of young (white columns) and elderly donors (black columns) with *p < 0.01.

We have shown that caspase-3 is implicated in the spontaneous and GM-CSF modulated apoptosis of PMN of young and elderly subjects. Caspase-3 is in an uncleaved form when not activated (procaspase-3). Here we analyzed by western-blotting experiments the amount of activated caspase-3 after different experimental conditions including GM-CSF and inhibitors treatment. We found that after 18 hours, GM-CSF-treated PMN of young subjects reduced significantly the expression of activated caspase-3 as compared to untreated cells (Figure 7A, young, lane 2 and 3, p < 0.01). In PMN of elderly subjects the expression of activated caspase-3 did no change after 18 hours of GM-CSF stimulation, compared to the non-stimulated status (Figure 7A, lane 2 and 3, elderly). When we applied the inhibitor PD98059, we found a reestablishment of the activated caspase-3 (Figure 7A lane 4, upper panel) compared to the GM-CSF-treated PMN. The p38 inhibitor, SB203580, did not influence significantly the activated caspase-3 expression modulation by GM-CSF. No modulation of the activated caspase-3 expression was found at any experimental conditions in PMN of elderly (Figure 7A lane 4, lower panel). These last data indicate a link between p42/p44MAPK and caspase-3 activation, however other transduction pathways could also play a role.

To confirm these results, we measured the caspase-3 activity by a specific fluorescent substrate (Table 2). In PMN of young subjects the activity of caspase-3 increased progressively after 6 hours of culture for reaching a peak value at 18 hours. For all the incubation times, the caspase-3 activity was significantly higher (p < 0.05) in the case of elderly donors (Table 2), except at 6 hours. Under GM-CSF stimulation, caspase-3 activity was significantly decreased at 6 hours (p < 0.05), 18 hours (p < 0.01) in PMN of young subjects. It is of note that GM-CSF could not restore the caspase-3 activity to the level of freshly prepared PMN. In the aged group, in PMN under GM-CSF treatment no significant decrease could be detected in caspase-3 activity compared to the spontaneous activities of caspase-3. These data support the fact that GM-CSF is involved in the modulation of caspase-3 activity which decreases in PMN of young subjects concomitantly to their rescue from apoptosis, while PMN of elderly subjects are unable to decrease caspase-3 activity when GM-CSF is provided in the milieu.

**Table 2:** Modulation of caspase-3 activity in PMN by MAPkinase inhibitors

Treatment	Culture time (hours)	Caspase-3 activity (arbitrary fluorescence units)	
		
		Young	Elderly
			
	0	92 ± 20	126 ± 29
			
None	6	218 ± 38	188 ± 53
	18	908 ± 118	1396 ± 207*
			
GM-CSF	6	113 ± 31	149 ± 42
	18	447 ± 87	979 ± 96*
			
PD98059	18	781 ± 216	1024 ± 413
			
GM-CSF + PD98059	18	1126 ± 376	1145 ± 387
			
SB203580	18	1055 ± 267	792 ± 291
			
GM-CSF + SB203580	18	459 ± 119	900 ± 147 *

PMN isolated from young and elderly donors were maintained in culture for the indicated periods of time. The cells were left untreated, or exposed to GM-CSF (20 ng/ml) or a combination of GM-CSF (20 ng/ml) and either in the presence or absence of PD98059 (30 μM) or SB203580 (10 μM). Caspase-3 activity was measured using a fluorescent substrate. Data are representative of 10 independent experiments. The asterisk (*) indicate significant differences (p < 0.05) between the two groups of donors.

To further link the MAPK pathways to survival/apoptosis of PMN we assessed the caspase-3 activity under GM-CSF stimulation after PD98059 and SB203580 pre-treatment. We found that any of these inhibitors alone did not modulate significantly the caspase-3 activity during the spontaneous apoptosis of PMN obtained either from young or elderly subjects (Table 2). The diminution of caspase-3 activity by GM-CSF in PMN of young subjects (from 908 ± 118 to 447 ± 87) could be completely reversed by PD98059 (from 447 ± 87 to 1126 ± 376), while the SB203580 could not modulate the effect of GM-CSF on caspase-3 activity. These inhibitors had no significant effect on PMN of elderly subjects. These data further confirm the essential role of the p42/p44 MAPK pathway in PMN survival by modulating the caspase-3 activity, while p38 MAPK is not concerned.

## Discussion

Many clinical data indicate that elderly subjects are more susceptible to infections, particularly to that of the higher respiratory tracts, more likely caused by atypical organisms as well as to sepsis by gram negative bacteria [9,14]. PMN are the first cells to arrive at the site of invasion and respond very quickly by the destruction of the aggressor. Recently, besides the alterations of the adaptive immune response it was demonstrated that some functions of the PMN including chemotaxis, killing and production of bactericidal substances are decreasing with aging [13] while others remain unchanged such as phagocytosis [41]. One particular aspect of neutrophils homeostasis is their propensity to die spontaneously i.e in the absence of pro-inflammatory stimuli for preserving the organism from undue destruction or chronic inflammation. In contrast, when an inflammatory process increases the level of pro-inflammatory mediators such as GM-CSF, G-CSF and LPS, PMN remain functional for 72 hours and die thereafter by apoptosis.

We have found that in PMN of elderly subjects the GM-CSF was not able to rescue them from apoptosis as efficiently as in PMN of young subjects [16]. Here we present data confirming and extending these results. Except GM-CSF any other agents used for modulating PMN apoptosis was found to demonstrate significant age-related differences. GM-CSF acts as a ligand for its specific receptor for exercising its anti-apoptotic effect. This means that if in PMN of elderly the GM-CSF is not able to exert its anti-apoptotic activity, an alteration in the signalling of GM-CSF may exist. In fact, in this study we demonstrate an alteration in the p42/p44 MAPK activation in PMN of elderly subjects participating in the altered rescue of PMN from apoptosis. Moreover, we show that p38 MAPK does not participate in the rescue from apoptosis either in young or elderly subjects. We present also data that molecules modulating the apoptotic fate of PMN were differentially related to the MAPK pathways. We demonstrate that the p42/p44 MAPK is not linked to the modulation of the expression of the Bcl-2 family members, while caspase-3 is partially modulated by this MAPK.

GM-CSF is activating three signalling pathways, Jak/STAT, MAPK and PI3K [19]. It is now well accepted that in eosinophils all three pathways are implicated in the GM-CSF induced rescue from apoptosis [42]. In PMN it is now also well established that p42/p44 MAPK and PI3K are implicated in the GM-CSF induced rescue from apoptosis [22]. We have recently shown that the Jak/STAT signalling pathway was also involved. We were the first to demonstrate that GM-CSF is unable to rescue PMN of elderly subjects from apoptosis [16]. This was since confirmed by other groups. This fact could have far reaching consequences on the susceptibility of elderly to infections. Thus it is very important to understand why this phenomenon is occurring in PMN of elderly.

As the p42/p44 MAPK was implicated in PMN of young subjects in the rescue from apoptosis, we studied whether this could contribute to the failure of rescue with aging. In fact, the activation of this MAPK by GM-CSF, as well as by other substances is strongly related to its anti-apoptotic effect, showed by the use of specific MEK1/2 inhibitors [26-30]. Our present data show that p42/p44 MAPK could not be activated by GM-CSF in the PMN of elderly. This inability exists either for the short stimulation times or for longer periods. It is clear from the experiments on PMN of young subjects that the activation of p42/p44 MAPK is sustained at least for 18 hours induced by GM-CSF. To confirm that the p42/p44 MAPK is participating to the rescue of PMN from apoptosis we used the inhibitor PD98059. We found, as others [26-30] that in PMN of young subjects the use of PD98059 reversed the GM-CSF inhibitor of apoptosis clearly demonstrating that the ERK/1/2 is effectively involved in the rescue of apoptosis. In the case of elderly subjects the lack of p42/p44 MAPK activation contributes to the altered rescue of PM from apoptosis and in fact, the PD98059 could not modulate the PMN apoptosis.

We also studied whether another member of the MAPK family namely the p38 MAPK participates to the GM-CSF induced rescue from apoptosis. The role of the p38 MAPK in PMN apoptosis is controversial [33-36]. Nevertheless, the consensus seems to exist that its activation participates in the PMN spontaneous apoptosis. Our present results confirm this contention in both age-groups. There is a controversy whether the activation of p38 MAPK participates in the rescue from apoptosis under various stimulations. In this regard the results of the literature seem to suggest that p38 MAPK does not participate in the PMN apoptosis delaying effect of GM-CSF [33-36]. Our present results indicate that p38 does not contribute to the rescue from apoptosis neither in PMN of young or elderly subjects.

Finally to understand how the p42/p44 MAPK can be implicated in the GM-CSF induced apoptosis of PMN we studied molecules intervening in the modulation of PMN apoptosis. These molecules enclosed those of the Bcl-2 family members and the caspases, mainly caspase-3. It was shown that the Bcl-2 family members are either pro-apoptotic or anti-apoptotic. In PMN the most important pro-apoptotic molecule is Bax and the most important anti-apoptotic ones are the Mcl-1, A1 and Bcl-xL [6]. The presence of Bcl-xL is still controversial. The ratio between these molecules determines the fate of PMN. We wanted to see whether the p42/p44 MAPK activation by GM-CSF is linked to the expression of the Bax and Bcl-xL molecules and their ratio. It is known that the p42/p44 MAPK is linked to the phosphorylation of Bad and freeing in this way the Bcl-xL, leading to an anti-apoptotic milieu [43]. We found that GM-CSF decreases the expression of Bax after 18 hours of stimulation, while it increased the expression of Bcl-XL which gives a ratio in favour of Bcl-xL, representing the survival. The contrary is occurring in PMN of elderly where the ratio is in favour of Bax and so pro-apoptotic. Weinmann *et al*. [44] showed that GM-CSF did not modulate the expression of Bcl-xL. Discrepancies may come from the fact that the concentration of GM-CSF used was 300 U/ml in their case and only 200 U/ml in our study. When we treated PMN with higher concentration we also found a drop in the effect of GM-CSF on Bcl-xL expression. The use of PD98059 could not reverted the effect of GM-CSF on the ratio of Bax/Bcl-xL indicating that the p42/p44 MAPK is not acting directly on these molecules of the Bcl-2 family. This indicates also that the p42/p44 MAPK is not the only signalling pathway participating in the GM-CSF induced rescue from apoptosis. As indicated the Jak/STAT and PI3K pathways are also participating. In PMN of elderly as the p42/p44 MAPK could not be activated by GM-CSF at any time points, it was evident that no modulation by ERK1/2 inhibitor was found. The p38 MAPK is not involved in the modulation of these molecules in PMN at any age-groups.

Furthermore, we were also interested to study the effects of p42/p44 MAPK on the expression and activity of another very important executioner protein, the caspase-3. Caspase-3 was implicated in the spontaneous and GM-CSF induced rescue of PMN from apoptosis. In fact we confirmed that GM-CSF could decrease the activated caspase-3 expression and activity in PMN of young subjects, while has no effect in PMN of elderly. It was also known that the p42/p44 MAPK is inhibiting the activation of caspase-8 [23]. The use of PD98059 indicated in PMN of young subjects that the p42/p44 MAPK is implicated in the GM-CSF induced inhibition of caspase-3, however this inhibition was not complete. This also indicates that the GM-CSF has other pathways to modulate the proteins participating in the executioner phase of apoptosis. In case of PMN of elderly subjects once again no modulation of caspase-3 could be demonstrated in any experimental conditions. For the participation of p38 MAPK in the modulation of caspase-3 by GM-CSF more work is needed as the results were not so clear cut.

Altogether, our data demonstrate to our best knowledge for the first time that the lack of activation of p42/p44 MAPK contribute to the decreased rescue of PMN from apoptosis in elderly individuals. This decreased activation results in a pro-apoptotic Bcl-2 family members over-expression and the activation of caspase-3 in contrast to PMN of young subjects. We also present data that the activation of p42/p44 MAPK should be sustained for at least 18 hours to ensure an efficient rescue of PMN from apoptosis by GM-CSF. Moreover, our results confirm that the p38 MAPK participates to the spontaneous apoptosis of PMN in both age-groups while it did not participate in the rescue of PMN apoptosis by GM-CSF. Thus, the perspective of modulation of the p42/p44 MAPK activation in PMN of elderly subjects may be useful to restore the effectiveness of GM-CSF and contribute to more effective PMN functionality in elderly. We explore actually what means can be used to achieve such an increase in p42/p44 MAPK activation, including the modification of the PMN membrane composition.
